# Turbinate Submucosal Reduction Operation Reduced Migraine Admission among Patients with Chronic Hypertrophic Rhinitis

**DOI:** 10.3390/ijerph17155455

**Published:** 2020-07-29

**Authors:** Chun-An Cheng, Yin-Han Chang, Chun-Gu Cheng, Hung-Che Lin, Chi-Hsiang Chung, Wu-Chien Chien

**Affiliations:** 1 Department of Neurology, Tri-Service General Hospital, National Defense Medical Center, Taipei 11490, Taiwan; cca@ndmctsgh.edu.tw; 2 Department of Psychology, National Taiwan University, Taipei 10621, Taiwan; caalice2003@yahoo.com.tw; 3 Department of Emergency Medicine, Taoyuan Armed Forces General Hospital, Taoyuan 32549, Taiwan; 4 Department of Emergency and Critical Medicine, Wan Fang Hospital, Taipei Medical University, Taipei 11696, Taiwan; 5 Department of Emergency Medicine, Tri-Service General Hospital, National Defense Medical Center, Taipei 11490, Taiwan; 6 Department of Otolaryngology-Head and Neck Surgery, Tri-Service General Hospital, National Defense Medical Center, Taipei 11490, Taiwan; lhj50702@gmail.com; 7 Department of Medical Research, Tri-Service General Hospital, National Defense Medical Center, Taipei 11490, Taiwan; g694810042@gmail.com; 8 School of Public Health, National Defense Medical Center, Taipei 11490, Taiwan; 9 Graduate Institute of Life Science, National Defense Medical Center, Taipei 11490, Taiwan

**Keywords:** migraine, chronic hypertrophic rhinitis, turbinate submucosal reduction operation

## Abstract

Rhinitis increases migraine risk. Chronic hypertrophic rhinitis can be treated with turbinate submucosal reduction operation. The relationship between migraine and chronic hypertrophic rhinitis after turbinate submucosal reduction operation is still unclear. The goal of this study was to evaluate the correlation between turbinate submucosal reduction operation and subsequent migraine admission in Asian chronic hypertrophic rhinitis patients. We identified patients suffering from chronic hypertrophic rhinitis and receiving turbinate submucosal reduction operation. The control group was selected from patients with chronic hypertrophic rhinitis without operation. The event was migraine admission. The risk factors of migraine admission were established using multivariate Cox proportional hazard regression. The risk of migraine admission after turbinate submucosal reduction operation is represented by a hazard ratio (HR) of 0.858 (95% confidence interval (CI): 0.633–0.962). The higher risk of migraine included depression with HR 4.348 (95% CI: 2.826–6.69), anxiety with HR 3.75 (95% CI: 2.267–6.203), fibromyalgia with HR of 7.326 (95% CI: 3.427–15.661), and asthma with HR 1.969 (95% CI: 1.11–3.491). Our study revealed that turbinate submucosal reduction operation led to a 14.2% reduction in migraine admission. Clinicians should understand the benefit of turbinate submucosal reduction operation and provide suitable treatments for comorbid conditions. Further prospective studies are required to confirm our findings.

## 1. Introduction

Migraine is a common disease throughout the world. Migraine headache is highly prevalent, and it impacts personal productivity, economy, and even national competitiveness. According to the Taipei community survey in Taiwan, it is increasing in prevalence, with 9.1% of people over 15 years of age suffering from migraine headache. Its overall prevalence is 4.5% in men and 14.4% in women [1].

Status migrainosus is defined as a debilitating migraine lasting longer than three days according to International Headache Society Diagnostic Criteria [2]. It should be addressed in an emergency setting with pain management [3]. Some severe headache patients should be treated with hospitalization for a short period. More than 15 days of headache every month is classified as chronic daily headache. If treatment is ineffective, withdrawal fails, or the condition is associated with other serious and complex medical or psychiatric illnesses, hospital-based treatment of headache is recommended [4].

Chronic rhinitis is defined as rhinitis that is persistent for 2 to 3 months. Rhinitis patients were found to suffer from comorbid conditions such as asthma, conjunctivitis, otitis media, sinusitis, eczema, food and insect bite allergies, migraine, and depression [5]. Chronic rhinitis may induce migraine due to inflammation, mast cell degranulation, and autonomic dysfunction [6]. Chronic hypertrophic rhinitis can cause nasal obstruction and interfere with breathing, and long-term nasal blockage can induce sleep apnea and possibly migraine headache [7]. Rhinitis represents inflammation of the nasal mucosa that affects work performance and quality of life and results in high health care costs. Immunotherapy for rhinitis can reduce migraine prevalence in younger women [8]. Chronic migraine patients have a higher incidence of rhinitis than episode migraine patients, but the incidence of rhinitis for other ethnicities was only 5.5% except for Caucasian or African-American patients [9]; population studies in Asians are limited. Studies about migraine and chronic hypertrophic rhinitis after turbinate submucosal reduction operation have provided unclear results. In this study, we investigated whether turbinate submucosal reduction operation could increase or reduce migraine admission for patients with chronic hypertrophic rhinitis. Previous studies about the prevalence of migraine in Taipei included only several thousand patients [1,10]; we used big data from the Taiwanese National Health Insurance Dataset to perform the study.

We used the inpatient database of the Taiwan Longitudinal National Health Insurance Database to retrieve cases of chronic hypertrophic rhinitis with or without turbinate submucosal reduction operation, followed by the outcome of migraine admission. Asian patients with chronic hypertrophic rhinitis underwent turbinate submucosal reduction operation to reduce migraine admission.

## 2. Materials and Methods

The Taiwan National Health Insurance (NHI), a single government payment insurance system, was implemented in 1995 and covers almost 99% of residents in Taiwan. The Taiwan National Health Insurance Database (NHIRD) contains the healthcare payment information of Taiwan. All medical facilities must submit computerized claims data for government payments. The data contain patients’ age, gender, facilities level, location, urbanization level, and admission time [11].

**Figure 1:**
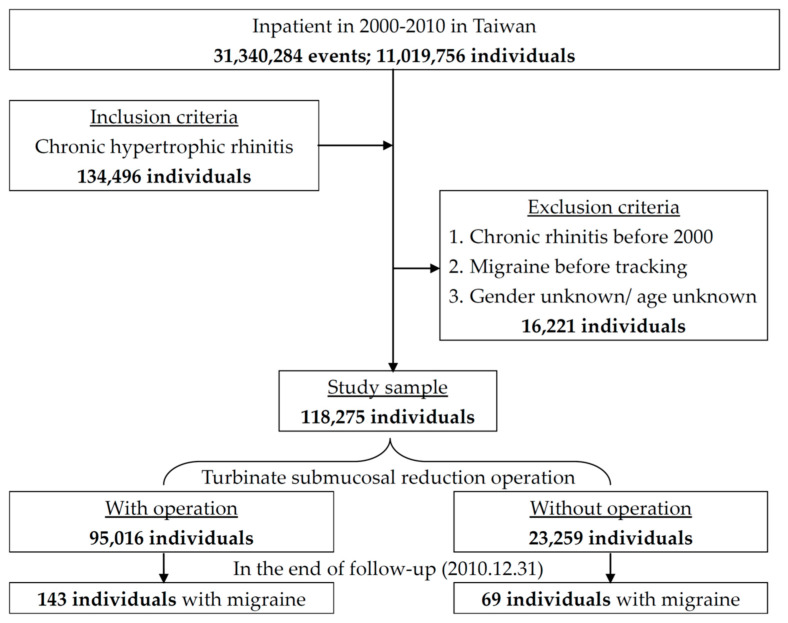
Flow chart of this study.

We designed a retrospective cohort study that used an inpatient dataset from 1 January 2000 to 31 December 2010. The data contained up to five diagnostic codes and five operation codes. The chronic hypertrophic rhinitis patients were retrieved with the International Classification of Disease, Ninth Revision, Clinical Modification (ICD-9-CM) operation code of 472.0 and turbinate submucosal reduction operation with an ICD-9-CM operation code of 21.6. Patients less than 18 years of age and those with unidentified gender, prior chronic hypertrophic rhinitis, or migraine diagnosed before 2000 were excluded. A previous study about the prevalence of migraine in Taipei found that the peak age was during 25–30 years old in males and 30–35 years old in females [10]. Because younger patients suffer from migraine more frequently, we divided patients into 3 age groups: less than 30 years old, between 30 and 45 years old, and more than 45 years old. The date of the first diagnosis of chronic hypertrophic rhinitis was defined as the index date. The outcome was defined as migraine admission (ICD-9-CM codes of 346) after the index date. The patients were followed until the first event or the end of this study. The study flowchart is shown in Figure 1. This study was approved by the Ethics Institutional Review Board of the Tri-Service General Hospital (TSGHIRB-2-104-05-126).

Comorbid conditions mapped by ICD-9-CM codes included diabetes mellitus (250), hypertension (401–405), hyperlipidemia (272), depression (296.2–296.3, 296.82, 330.4, 331), anxiety (300.1–300.3, 300.5–300.9), chronic fatigue syndrome (780.7), fibromyalgia (729.1), irritable bowel syndrome (564.1), sleep disturbance (307.4, 780.5), temporomandibular joint disorder (524.6), dysmenorrhea (625.3), and asthma (493).

Descriptive statistics were computed to analyze the turbinate submucosal reduction operation and non-turbinate submucosal reduction operation groups. Student’s t-test was used for continuous variables, and the chi-squared (X^2^) test was used for categorical variables. The difference between the cumulative incidences of the two groups was assessed with the log-rank test. The risk factors for migraine admission were identified using a multivariable Cox proportional hazard regression model. A *p*-value < 0.05 was considered to indicate statistical significance. The risk of migraine admission was presented as a hazard ratio (HR) after adjustment for the season, area, urbanization level, hospital type, and income. All statistical analyses were performed using SPSS software version 21 (International Business Machine Company, Armonk, NY, USA).

## 3. Results

**Figure 2:**
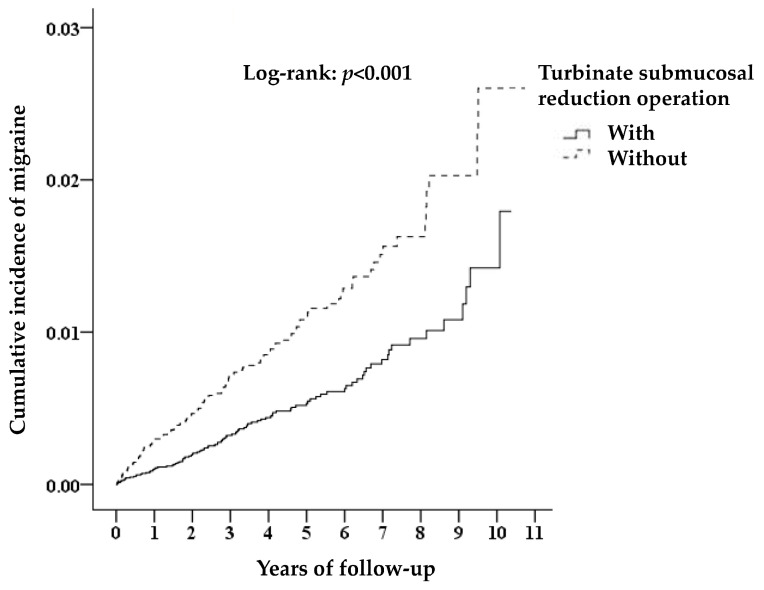
Kaplan–Meier analysis of the cumulative risk of migraine among chronic hypertrophic rhinitis patients stratified by turbinate submucosal reduction operation with the log-rank test.

A total of 118,275 chronic hypertrophic rhinitis patients were admitted from 2000–2010; 95,016 who underwent turbinate submucosal reduction operation were assigned to the case group, and 23,259 without surgery were assigned to the control group. The chronic hypertrophic rhinitis patients receiving turbinate submucosal reduction operation were associated with a reduced risk of migraine admission. The case selection and outcome follow-up are shown in Figure 1. There was a statistically significant difference in migraine admission between the two groups in long-term follow-up; 0.15% (143/95016) of the case group were admitted due to migraine compared with 0.3% (69/23259) of the control group (log-rank *p* < 0.001) (Figure 2).

Male gender, younger age, and less comorbidity were common in those who received turbinate submucosal reduction operation. Chronic hypertrophic rhinitis patients with higher levels of urbanization and insured premiums who lived in northern Taiwan tended to undergo surgery in the summer. Most turbinate submucosal reduction operations were performed in medical centers. The characteristics of the different groups are described in Table 1.

The risk factors for migraine admission with multivariate analyses are shown in Table 2. After adjusting for age, gender, and comorbidities, chronic hypertrophic rhinitis patients who underwent turbinate submucosal reduction operation had a reduced risk of severe migraine admission with a hazard ratio of 0.858 (95% CI: 0.633–0.962) (*p* = 0.022). The male chronic hypertrophic rhinitis patients had a lower risk of subsequent migraine admission than females (HR 0.603, 95% CI: 0.458–0.794) (*p* < 0.001). The other increasing risk factors for migraine admission were depression (HR 4.348, 95% CI: 2.826–6.69) (*p* < 0.001), anxiety (HR 3.75, 95% CI: 2.267–6.203) (*p* < 0.001), fibromyalgia (HR 7.326, 95% CI: 3.427–15.661) (*p* < 0.001), and asthma (HR 1.969, 95% CI: 1.11–3.491) (*p* = 0.02). We surveyed the effect of medications in surgical patients, and found that beta blockers (HR 0.863; 95% CI: 0.726–0.95, *p* = 0.007), flunzrizine (HR 0.912; 95% CI: 0.824–0.998, *p* = 0.049), and antiepileptic agents including volproic acid or topiramate (HR 0.668; 95% CI: 0.531–0.774, *p* < 0.001) reduced migraine admission.

## 4. Discussion

Turbinate submucosal reduction operation prevents migraine disability in chronic hypertrophic rhinitis patients, reducing the social burden. The operation seeks to remove inflammation and improve hypoxia, which is essential to reduce migraine admission. Comprehensive knowledge about migraine and aggressive surgical treatment of chronic hypertrophic rhinitis is important for physicians in these two related diseases. Approximately 78% of asymptomatic migraine patients underwent successful siniorhinological surgery in a small sample study [12]. Migraine is an expression of central neuronal hyperexcitability. To reduce migraine attacks, patients should decrease possible migraine triggers and avoid factors that induce a migraine attack, such as poor sleep, malaise, pressure, certain foods, and vasodilators. In addition, migraine is an estrogen-sensitive disorder, with oral contraceptives and hormone therapy as possible triggers, requiring the modification, reduction, or cessation of dosages. The symptoms of migraine occur through the trigeminal–autonomic reflex. Pain afferents descend to the caudal trigeminal nucleus and dorsal horns of the upper cervical spinal cord. Dysfunction of the serotonergic antinociceptive nuclei, which provide descending inhibitory control of sensory neurons of the caudal trigeminal nucleus, could facilitate the trigeminal nociceptive system [13]. The facilitation of the trigeminal nociceptive system is a unique characteristic of migraine headache, but is not present in acute sinusitis [14]. Almost 2% of the general population have chronic migraine; there is a significant socioeconomic impact on society, such as increased health care costs and decreased quality of life. The management of these headache patients should be a priority [15].

Approximately 10.8% of patients had a migrainous chief complaint in otolaryngology outpatient practice [16]. Almost 90% of sinus headache patients were determined to have migraine. Sinus pain, sinus pressure, nasal symptoms, and ocular symptoms are features of migraine [17]. Sinus headache and migraine are easily confused with one another. Approximately 86% of self-diagnosed sinus headaches have migraine or probable migraine, and 75% of migraines presented cranial autonomic symptoms during headache attack [18,19]. The aforementioned study focused on rhinitis symptoms and comorbid conditions according to patients’ self-reports.

Hypoxia induces the production of nitric oxide in nasal and paranasal mucosa, increasing oxygen absorption, and nitric oxide induces the nerve impulses of the trigeminal sensory nerve to cause cortical spreading depression and vasodilation [20]. Hypoxic nitric oxide is an additional pathological feature of migraine [21].

Allergy test-positive patients had a higher frequency of migraine attacks [22]. Rhinitis is a histamine-driven syndrome that can affect the central nervous system. Allergic rhinitis through histamine triggers migraine headache attacks by vasodilation with nitric oxide release and inflammation with mast cell degranulation [23]. Inflammatory mediators with vasoactive function are important in migraine and rhinitis. Adolescent migraine is associated with asthma or seasonal allergies of inflammatory conditions [24]. Allergens can influence trigeminal afferents through the enhanced release of inflammatory mediators with degranulation from dural mast cells. Rhinitis was found to be associated with headache frequency (adjusted OR 1.25, 95% CI 1.05–1.49) and headache-related disability (adjusted OR 1.10, 95% CI 0.96–1.26) [6]. A past study found that immunotherapy administration decreased the prevalence, frequency, and disability of migraine sufferers in younger patients compared with 45-year-old allergic patients [8].

More male chronic hypertrophic rhinitis patients underwent surgery in our study, with potential greater severity; however, the risk of migraine admission was greater for females than for males. Younger patients, those living in northern Taiwan, those with high income, and those with fewer comorbid conditions tended to undergo the operation to improve their quality of life. The majority of patients received the operation in medical centers with adequate facilities to handle complications from the operation such as nasal bleeding, septal perforation, olfactory dysfunction, and cerebrospinal fluid leakage.

Migraine, rhinitis, and back problems are related to major depression [25]. Depression and anxiety induce the migraine admission of chronic hypertrophic rhinitis patients. Norepinephrine and serotonin inhibit central excitability, but low norepinephrine and serotonin levels were noted in patients with depression. Serotonin deficiency increases painful sensation in chronic pain [26], and low norepinephrine was noted in migraine [27]. Migraine with psychological syndrome increases symptom severity and requires hospital treatment. More psychological symptoms increased migraine admission in our study. Fibromyalgia carried a risk of migraine with adjusted HR of 1.89 (95% CI: 1.75–1.96) [28]; our study found a higher risk, with the potential explanation that depression reduced pain inhibition. A previous study showed asthma to be related to the occurrence of chronic migraine with an adjusted odds ratio of 2.01 [29]. Our study showed a similar result. Anti-asthmatic or anti-allergic therapies were associated with a decreased risk of migraine in children and adolescents [30].

A greater benefit was found in female chronic hypertrophic rhinitis patients who underwent the operation (adjusted HR 0.77; 95% CI: 0.51–0.862) in stratified analysis, because there was a higher prevalence of migraine in younger females. This caused male patients to carry a lower risk of migraine. Diabetes mellitus patients have a lower risk of migraine admission because diabetes mellitus with polyneuropathy induces poor sensation. The operation was more effective in patients 30–45 years old (adjusted HR 0.649; 95% CI: 0.398–0.959) in stratified analysis. The majority, around 77% of patients with chronic hypertrophic rhinitis, received the operation below 45 years of age, and no relationship was shown between age and migraine. Hypertension and hyperlipidemia were treated according to diagnostic codes, with adequate treatment resulting in no association with migraine. Migraine and irritable bowel syndrome showed rates of recurrent pain and female predominance. Serotonin acts as an inhibitory mediator between the central nervous system and the enteric nervous system. A previous study revealed a similarly increased risk (HR 1.95) of irritable bowel syndrome in the migraine group compared with the migraine-free group in an Asian population [31]. The present study found that irritable bowel syndrome was not related to migraine admission due to lower prevalence. Sleep apnea is a potential link between nasal blockage that induces apnea and migraine headache, as nasal blockage can worsen sleep apnea and possibly migraine headache [7]. The operation improved the nasal obstruction and sleep quality. Chronic hypertrophic rhinitis patients with sleep disorders who received the operation showed a reduced risk of migraine admission (HR: 0.186, 95% CI: 0.049–0.7, *p* = 0.013) in stratified analysis. The potential reason could be that the operation resolved nasal problems greater than sleep disturbance, which led to a decline in migraine admission.

This study included a big sample of 110,000 patients with chronic hypertrophic rhinitis, who freely selected the operation, though most payments were made by national health insurance; the operation promoted patients’ health outcomes. The strength of this study was that it included Chinese patients with chronic hypertrophic rhinitis who received operations in a nationwide population-based cohort study, which represents the whole population of Taiwan. There were several limitations in our study. First, because the NHIRD is a payment claims dataset, there were no detailed data on body mass index, family history, electroencephalography studies, and severity of migraine; we could not determine whether hypoxic change or turbinate submucosal reduction operation could reduce the degree of pain in migraine. Second, patients with advanced age and more comorbid conditions treated non-surgically had more migraine admissions; they should be studied with matched patients with turbinate submucosal reduction operation. Third, we investigated chronic hypertrophic rhinitis patients in a Chinese population. Future prospective studies of other ethnic groups are needed to validate our findings.

## 5. Conclusions

To our knowledge, this is the first paper to survey chronic hypertrophic rhinitis patients who received turbinate submucosal reduction operation to reduce the risk of migraine admission. The aggressive management of psychological disorders, fibromyalgia, and asthma in female chronic hypertrophic rhinitis patients reduced migraine severity. A further prospective study needs to be performed to confirm our findings.

## Figures and Tables

**Table 1:** Characteristics of the patients at baseline.

Turbinate Submucosal Reduction Operation	Total		With		Without		*p*
Variables	*n*	%	*n*	%	*n*	%	
	118,275		95,016	80.33	23,259	19.67	
**Gender** (Male)	83,307	70.44	68,443	72.03	14,864	63.91	<0.001
**Age (years)**	37.58 ± 14.28		35.56 ± 12.78		45.85 ± 16.87		<0.001
**Age group (years)**							<0.001
<30	44,678	37.77	39,810	41.90	4868	20.93	
30–45	40,317	34.09	33,320	35.07	6997	30.08	
≥45	33,280	28.14	21,886	23.03	11,394	48.99	
**Diabetes mellitus**	2093	1.77	851	0.90	1242	5.34	<0.001
**Hypertension**	4348	3.68	2159	2.27	2189	9.41	<0.001
**Hyperlipidemia**	356	0.30	104	0.11	252	1.08	<0.001
**Depression**	404	0.34	103	0.11	301	1.29	<0.001
**Anxiety**	400	0.34	85	0.09	315	1.35	<0.001
**Chronic fatigue syndrome**	2	0.00	1	0.00	1	0.00	0.355
**Fibromyalgia**	73	0.06	10	0.01	63	0.27	<0.001
**Irritable bowel syndrome**	27	0.02	8	0.01	19	0.08	<0.001
**Sleep disturbance**	6052	4.93	4457	4.48	1595	6.86	<0.001
**Temporomandibular joint disorder**	12	0.01	2	0.00	10	0.04	<0.001
**Dysmenorrhea**	4	0.00	1	0.00	3	0.01	0.026
**Asthma**	1804	1.53	352	0.37	1452	6.24	<0.001
**Season**							<0.001
Spring (March–May)	31,062	26.26	24,846	26.15	6216	26.73	
Summer (June–August)	30,437	25.73	24,789	26.09	5648	24.28	
Autumn (September–November)	27,952	23.63	22,340	23.51	5612	24.13	
Winter (December–February)	28,824	24.37	23,041	24.25	5783	24.86	
**Location**							<0.001
Northern Taiwan	47,644	40.28	40,118	42.22	7526	32.36	
Middle Taiwan	34,757	29.39	26,919	28.33	7838	33.70	
Southern Taiwan	31,678	26.78	25,792	27.14	5886	25.31	
Eastern Taiwan	4070	3.44	2095	2.20	1975	8.49	
Outlet islands	126	0.11	92	0.10	34	0.15	
**Urbanization level**							<0.001
1 (Highest)	44,393	37.53	37,016	38.96	7377	31.72	
2	57,057	48.24	46,318	48.75	10,739	46.17	
3	5222	4.42	3549	3.74	1673	7.19	
4 (Lowest)	11,603	9.81	8133	8.56	3470	14.92	
**Level of care**							<0.001
Medical center	55,660	47.06	47,288	49.77	8372	35.99	
Regional hospital	51,281	43.36	38,961	41.00	12,320	52.97	
Local hospital	11,334	9.58	8767	9.23	2567	11.04	
**Insured premium (NT$)**							<0.001
<18,000	115,477	97.63	92,717	97.58	22,760	97.85	
18,000–34,999	1748	1.48	1399	1.47	349	1.50	
35,000+	1050	0.89	900	0.95	150	0.64	

**Table 2:** Risk factors of migraine at the end of follow-up according to Cox regression.

Variables	Crude HR	95% CI	95% CI	*p*	Adjusted HR	95% CI	95% CI	*p*
**Turbinate Submucosal Reduction Operation**								
	0.759	0.569	0.911	0.011 *	0.858	0.633	0.962	0.022 *
**Gender**								
**Male**	0.521	0.398	0.682	<0.001 *	0.603	0.458	0.794	<0.001 *
**Female**	Reference				Reference			
**Age (years)**								
<30	Reference				Reference			
30–45	1.569	1.018	2.501	0.041 *	1.479	0.942	2.324	0.089
≥45	1.361	0.877	2.111	0.169	1.276	0.803	2.029	0.302
**Diabetes mellitus**	0.531	0.289	0.975	0.041 *	0.479	0.255	0.9	0.022 *
**Hypertension**	1.079	0.750	1.553	0.680	1.366	0.919	2.029	0.123
**Hyperlipidemia**	1.148	0.566	2.329	0.701	1.028	0.493	2.143	0.941
**Depression**	7.560	5.158	11.082	<0.001 *	4.348	2.826	6.69	<0.001 *
**Anxiety**	6.398	4.418	10.894	<0.001 *	3.750	2.267	6.203	<0.001 *
**Chronic fatigue syndrome**	10.813	1.515	77.160	0.018 *	3.882	0.500	30.124	0.195
**Fibromyalgia**	13.303	8.538	27.070	<0.001 *	7.326	3.427	15.661	<0.001 *
**Irritable bowel syndrome**	6.849	1.682	27.881	0.007 *	3.368	0.800	14.182	0.098
**Sleep disturbance**	2.568	1.465	4.503	0.001 *	1.711	0.954	3.067	0.071
**Asthma**	2.226	1.270	3.901	0.005 *	1.969	1.11	3.491	0.02 *
**Season**								
Spring (March–May)	Reference				Reference			
Summer (June–August)	0.957	0.637	1.439	0.834	0.941	0.626	1.416	0.772
Autumn (September–November)	1.097	0.748	1.609	0.636	1.042	0.709	1.532	0.832
Winter (December–February)	1.225	0.822	1.825	0.319	1.198	0.801	1.787	0.381
**Urbanization level**								
1 (Highest)	0.765	0.494	1.185	0.231	0.970	0.603	1.560	0.900
2	1.139	0.776	1.674	0.507	1.409	0.941	2.111	0.096
3	0.940	0.525	1.683	0.835	1.037	0.578	1.863	0.902
4 (Lowest)	Reference				Reference			
**Level of care**								
Medical center	0.685	0.470	0.997	0.048 *	0.672	0.445	1.015	0.059
Regional hospital	0.873	0.621	1.228	0.438	0.825	0.583	1.168	0.278
Local hospital	Reference				Reference			
**Insured premium (NT$)**								
<18,000	Reference				Reference			
18,000–34,999	1.667	0.740	3.754	0.217	1.799	0.797	4.063	0.158
35,000+	0.691	0.097	4.927	0.712	0.695	0.125	6.399	0.912

* *p* < 0.05; HR: hazard ratio; CI: confidence interval
